# Segmental Variation in Lumbar Nerve Root Weight: A Donor-Body Study

**DOI:** 10.7759/cureus.116139

**Published:** 2026-09-12

**Authors:** Michael E Pollack, Sunny Patel, Venkatasaicharan Dyavanapalli, Matthew Chan, James Adams, Shanu Markand

**Affiliations:** 1 Anatomy, A.T. Still University, Kirksville, USA; 2 Diagnostic Radiology, A.T. Still University, Kirksville, USA

**Keywords:** cauda equina, cord compression, donor body, lumbar spinal cord, nerve root weight

## Abstract

Introduction

The cauda equina consists of lumbosacral nerve roots extending from the conus medullaris within the lumbar cistern. Although radiographic imaging is essential for evaluating lumbar spinal pathology, normal anatomical variation in the cauda equina may affect its appearance. This donor-body study quantified normalized segmental linear mass per root across the L1-L2 through L4-L5 vertebral intervals and evaluated level-specific and demographic patterns. These measurements provide exploratory anatomical data for future imaging and clinical correlation studies.

Methods

Thirty-two donor bodies underwent posterior lumbar dissection from L1 through L5. The craniocaudal length of each lumbar interval was measured using vertebral body borders as standardized anatomical endpoints. The dura and neural contents were removed en bloc, after which the dura and all visible connective tissue were removed. The isolated neural tissue was transected at the corresponding vertebral body borders, allowed to become free of visible surface moisture, and weighed in milligrams. Normalized segmental linear mass per root was calculated by dividing the segment mass by its measured length and the estimated mean number of root components traversing that interval.

Results

Normalized segmental linear mass per root differed significantly across lumbar levels (Friedman χ²(3) = 24.30, p = 2.16 × 10⁻⁵). The highest median value was observed at L1-L2 (1.42 × 10⁻³ mg/mm per root), and the lowest was observed at L4-L5 (0.97 × 10⁻³ mg/mm per root). After Bonferroni correction, L4-L5 values were significantly lower than those at L1-L2, L2-L3, and L3-L4. No association with age, height, body weight, or sex remained statistically significant after false discovery rate (FDR) correction.

Conclusions

Normalized segmental linear mass per root generally decreased caudally and was lowest at L4-L5. No demographic association remained statistically significant after correction for multiple comparisons. These findings provide exploratory anatomical data regarding level-specific variation within the cauda equina; their imaging, procedural, and clinical implications require validation through studies directly correlating anatomic measurements with imaging and clinical outcomes.

## Introduction

Anatomy

The spinal cord is a vital component of the central nervous system. The majority of the spinal cord is entirely surrounded by the bony vertebral column, typically beginning at the inferior-most portion of the medulla oblongata in the brain and terminating at the level of the first or second lumbar vertebrae, where it tapers to form the end of the spinal cord, known as the conus medullaris [1]. The spinal cord is typically covered by three meningeal layers: the pia, arachnoid, and dura mater, which surround it until its termination [2]. After this occurs, the dura and arachnoid mater continue to form the lumbar cistern, which typically extends from L2-S2 [3]. Contained within the lumbar cistern are three major components: cerebrospinal fluid, the distal extension of the pia mater known as the filum terminale, and the cauda equina [4].

The cauda equina is a collection of nerve roots that originate from the conus medullaris. These nerve roots are responsible for sensory and motor function of the lower extremities, as well as bladder, bowel, and genital function [5].

General pathology

Lumbar spine pathologies are among the most common issues encountered by medical professionals [6,7]. The lifetime prevalence of lumbar spine pain ranges from 30% to 80% worldwide [6]. The majority of lumbar spine pain is caused by soft tissue injury and is associated with age, genetics, and mechanical stress [7]. Lumbar spinal pathologies may arise from anatomic or physiologic changes in response to physical activity, sedentary lifestyle, and genetic predisposition [8].

Anatomical pathology

The lumbar spine is susceptible to anatomic pathologies. One common pathological condition is lumbar disc herniation [9]. Another pathology in this category is a spinal compression fracture, where vertebral body collapse may result in vertebral retropulsion into the spinal canal [10]. Scoliosis is characterized by abnormal lateral curvature of the spine and may lead to spinal stenosis or asymmetric compression of spinal nerve roots [11]. A common theme among these anatomical pathologies is their potential to increase vulnerability of nerve roots within the spinal canal to compression [12]. For example, in lumbar disc herniation, displacement of disc material may encroach upon the spinal canal, increasing the likelihood of neurologic symptoms [9].

Physiologic and clinical pathology

One of the most emergent but infrequent causes of lower back pain is cauda equina syndrome [5,13]. This condition is typically caused by severe spinal stenosis, leading to compression of the cauda equina within the lumbar cistern and resulting in severe radicular pain, saddle anesthesia, and bowel or bladder dysfunction. Cauda equina syndrome is a neurologic emergency and, if left untreated, may result in permanent neurologic deficits [5,14,15].

Cauda equina syndrome is a clinical diagnosis and may be associated with multiple comorbidities that increase mechanical load on the lumbar spine, including obesity [5] and poor posture [15]. Certain physiologic and pathologic states, such as pregnancy or gynecologic malignancy, may exacerbate underlying lumbar pathology and contribute to compression risk [15]. In these cases, compression may occur through mass effect or, in rare instances, leptomeningeal spread of malignancy [16].

Clinical implications

The lumbar region also serves as the primary access site to the spinal canal for diagnostic and therapeutic procedures, including epidural and spinal anesthesia, lumbar drains, steroid injections, and lumbar puncture [17]. Among these, lumbar puncture and epidural injections are the most common procedures with significant clinical implications [18]. Lumbar punctures for the general population are typically performed at the L3-L4 or L4-L5 interspaces to minimize the risk of spinal cord injury [19]. Extensive radiographic and clinical research supports the use of these interspaces as the safest sites for lumbar puncture [20].

Although imaging techniques are accurate and considered the standard of care, they may identify findings that reflect normal anatomic variation rather than true pathology. Processes such as arachnoiditis or apparent spinal stenosis may mimic other pathologies [21]. Donor body studies provide a unique opportunity to explore structural pathways of vulnerability to compression syndromes [22]. Unlike imaging studies, donor body investigations allow for direct anatomic visualization and measurement of nerve roots, their spatial distribution within the dural sac, and their relationships within the spinal canal [23].

Existing literature suggests that spinal canal compromise is often attributed to reduced canal diameter or increased external compression from disc material, ligamentous hypertrophy, or other mass effects [12]. As a result, much of the literature has focused on thecal sac caliber. Previous anatomical studies have characterized the organization, diameter, cross-sectional area, rootlet number, and imaging appearance of cauda equina nerve roots [24-26]. Hogan used nerve root mass and length to estimate individual root cross-sectional area, while Wall et al. and Arslan et al. described intrathecal organization, root diameter, and rootlet number [24-26]. However, we identified no prior study reporting normalized segmental linear mass per root across sequential L1-L5 vertebral intervals.

The purpose of this study was to quantify normalized segmental linear mass per root in sequential segments of the cauda equina from superior to inferior. We also evaluated whether these measurements varied by lumbar level, sex, age, height, and body weight. By mapping level-specific and demographic patterns, this study aimed to provide an anatomic framework for future imaging and clinical correlation studies.

We hypothesized that normalized segmental linear mass per root would vary systematically by lumbar level and sex, with decreasing values in a caudal direction and distinct patterns between males and females. This study did not directly evaluate imaging findings, compression, procedural safety, or clinical outcomes.

## Materials and methods

The A.T. Still University Institutional Review Board reviewed the study and determined it to be exempt in accordance with institutional protocols (protocol no. SM20250820-001). The study included 32 donor bodies obtained through the institution’s anatomical donation program, comprising 18 (56.3%) male and 14 (43.8%) female donor bodies aged 43-95 years as shown in Table 1. Because this was an exploratory donor-body study and no published estimates of the primary level-specific outcome were available for effect-size estimation, an a priori power calculation was not performed. The sample size was determined by the number of donor bodies available for dissection during the study period.

**Table 1:** Demographic and clinical characteristics of the donor bodies The study included 32 donor bodies: 18 (56.3%) male and 14 (43.8%) female. The mean age was 77.1 ± 11.6 years (range, 43-95 years).

Donor body (assigned number)	Sex	Age (years)	First-listed recorded cause of death
1	Male	73	Intrahepatic cholangiocarcinoma
2	Female	93	Congestive heart failure
3	Male	90	Dementia
4	Female	82	End-stage renal disease
5	Male	64	Unknown
6	Female	84	Congestive heart failure
7	Male	87	Acute hypoxic respiratory failure
8	Female	63	Cardiac arrest
9	Male	65	Chronic obstructive pulmonary disease
10	Male	43	Hypoxic shock
11	Male	82	Cardiopulmonary arrest
12	Female	78	Respiratory failure
13	Male	63	Pancreatic cancer
14	Female	77	Malignant neoplasm of the ampulla of Vater
15	Male	82	Malignant neoplasm of the pancreatic tail
16	Female	78	Atherosclerotic heart disease
17	Male	74	Metabolic encephalopathy
18	Male	62	Complications of alcohol abuse
19	Male	89	Bladder cancer
20	Female	73	Bipolar depression
21	Male	60	Metastatic malignant neoplasm of the colon
22	Female	85	Centrilobular emphysema
23	Male	73	Cardiovascular cause, unspecified
24	Female	95	Cardiopulmonary arrest
25	Male	76	Pneumonia
26	Female	81	Acute respiratory failure
27	Male	79	Multiple myeloma
28	Female	89	Metastatic colon cancer
29	Male	70	Acute-on-chronic respiratory failure
30	Female	91	Cerebral atherosclerosis
31	Male	88	Late effects of stroke
32	Female	78	Cardiac cause, unspecified

The posterior lumbar region of L1-L5 was carefully dissected to access the spinal canal. A scalpel was used to dissect through skin, fascia, musculature, and ligaments to reach the laminae, which were then removed with a hammer and chisel. The laminectomy from the gross anatomy dissection was identified and extended to provide greater exposure of the spinal canal and localize the L1-L5 vertebrae. Each lumbar region was photographed with an iPhone 16 Pro Max (Apple Inc., Cupertino, CA, USA; 48 MP main camera) under consistent lighting to ensure high-detail documentation.

Spinal canal measurements

The full craniocaudal span analyzed extended from the superior border of L1 to the inferior border of L5 and was divided into four consecutive lumbar intervals: L1-L2, L2-L3, L3-L4, and L4-L5. The craniocaudal length of each interval was measured using the corresponding vertebral levels as anatomical landmarks. Measurements were obtained with a LOUISWARE Silver Digital Caliper (0-150 mm range; 0.01 mm resolution; ±0.02 mm accuracy; ASIN B087N5N45G). Using blunt dissection, the dura mater and neural contents were removed en bloc from the superior border of L1 through the inferior border of L5. After removal from the spinal canal, the dura mater and all visible connective tissue were carefully dissected away so that only neural tissue remained. The specimen was maintained in its original rostral-to-caudal orientation for segmentation, as shown in Figure 1.

**Figure 1:**
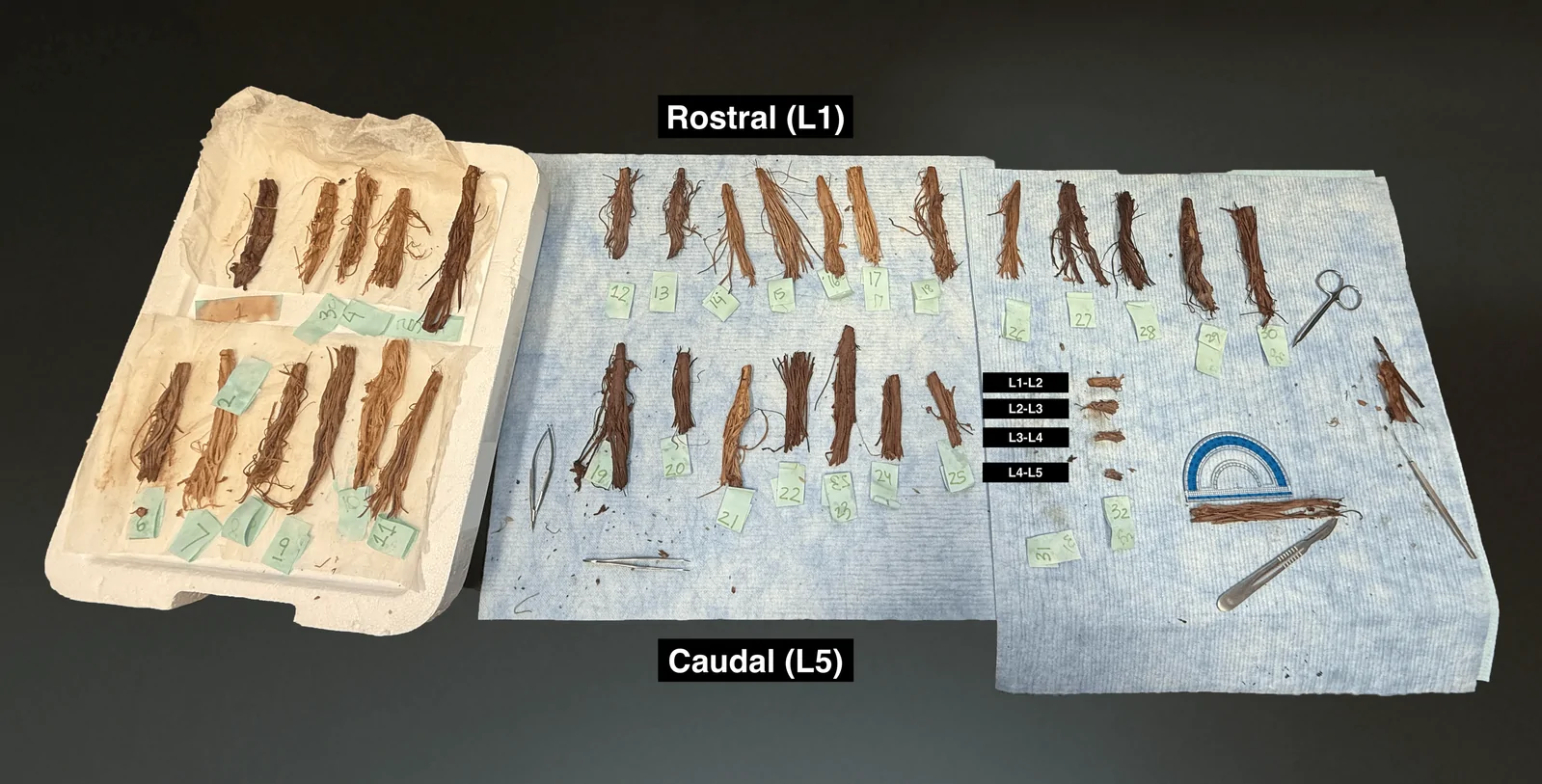
Lumbar neural specimens following spinal canal dissection Lumbar neural specimens following spinal canal dissection. Display of the 32 lumbar neural specimens following removal from the spinal canal and excision of the dura mater. The separated specimen on the right demonstrates the four analyzed lumbar intervals from L1-L2 through L4-L5.

Segmental neural tissue preparation and weighing

After the dura mater was removed, all visible connective tissue was carefully dissected away so that only isolated neural tissue remained. The neural tissue was transected transversely at the same vertebral body borders used to define the L1-L2, L2-L3, L3-L4, and L4-L5 intervals. Individual nerve roots were not separately dissected to a uniform length. Instead, each composite neural segment corresponded to the measured craniocaudal length of its respective vertebral interval, and the subsequent calculation normalized its mass to that measured length. The segments were allowed to become free of visible surface moisture before weighing. Each segment was weighed in milligrams using a Sartorius ENTRIS124-1S balance (Sartorius Lab Instruments GmbH & Co. KG, Göttingen, Germany; capacity, 120 g; readability, 0.1 mg).

Statistical analysis

Normalized segmental linear mass per root was calculated as: $\frac{\text{Segment neural tissue mass (mg)}}{\text{Segment length (mm)} \times \text{Estimated mean root count}}$.

Estimated mean root counts were 42, 38, 34, and 30 for L1-L2, L2-L3, L3-L4, and L4-L5, respectively. These values represented the mean estimated number of bilateral dorsal and ventral root components at the boundaries of each interval. The mass-to-length calculation was informed by Hogan [26], while normalization by estimated root count was developed for this study.

Descriptive statistics were calculated for each lumbar level, and normality was assessed using the Shapiro-Wilk test. Because measurements were repeated within the same donor bodies and were not normally distributed at every level, differences across levels were evaluated using the Friedman test, followed by Wilcoxon signed-rank tests with Bonferroni correction. Paired Cohen’s d was reported as an absolute measure of effect size. Sex differences were evaluated using the Mann-Whitney U test, while associations with age, height, and body weight were evaluated using Spearman correlations. Benjamini-Hochberg false discovery rate (FDR) correction was applied separately within each demographic analysis. Statistical significance was defined as p < 0.05. Analyses were performed using Microsoft Excel (Microsoft Corporation, Redmond, WA, USA).

## Results

Thirty-two donor bodies were included in the final analysis: 18 (56.3%) male and 14 (43.8%) female. Normalized segmental linear mass per root generally decreased caudally. The highest median value was observed at L1-L2 (1.42 × 10⁻³ mg/mm per root), and the lowest was observed at L4-L5 (0.97 × 10⁻³ mg/mm per root). Descriptive statistics by lumbar level are presented in Table 2, and their distributions are shown in Figure 2.

**Table 2:** Normalized segmental linear mass per root across lumbar levels Normalized segmental linear mass per root was calculated for 32 donor-body specimens at each lumbar level. Values are reported as median (IQR), mean ± SD, and minimum-maximum. IQR, interquartile range; SD, standard deviation

Lumbar level	Median (IQR) (×10⁻³ mg/mm per root)	Mean ± SD (×10⁻³ mg/mm per root)	Min-Max (×10⁻³ mg/mm per root)
L1-L2	1.42 (1.26-1.67)	1.50 ± 0.42	0.85-2.70
L2-L3	1.37 (1.11-1.84)	1.50 ± 0.64	0.61-3.90
L3-L4	1.17 (0.98-1.54)	1.30 ± 0.52	0.60-3.00
L4-L5	0.97 (0.51-1.54)	1.10 ± 0.64	0.13-2.30

**Figure 2:**
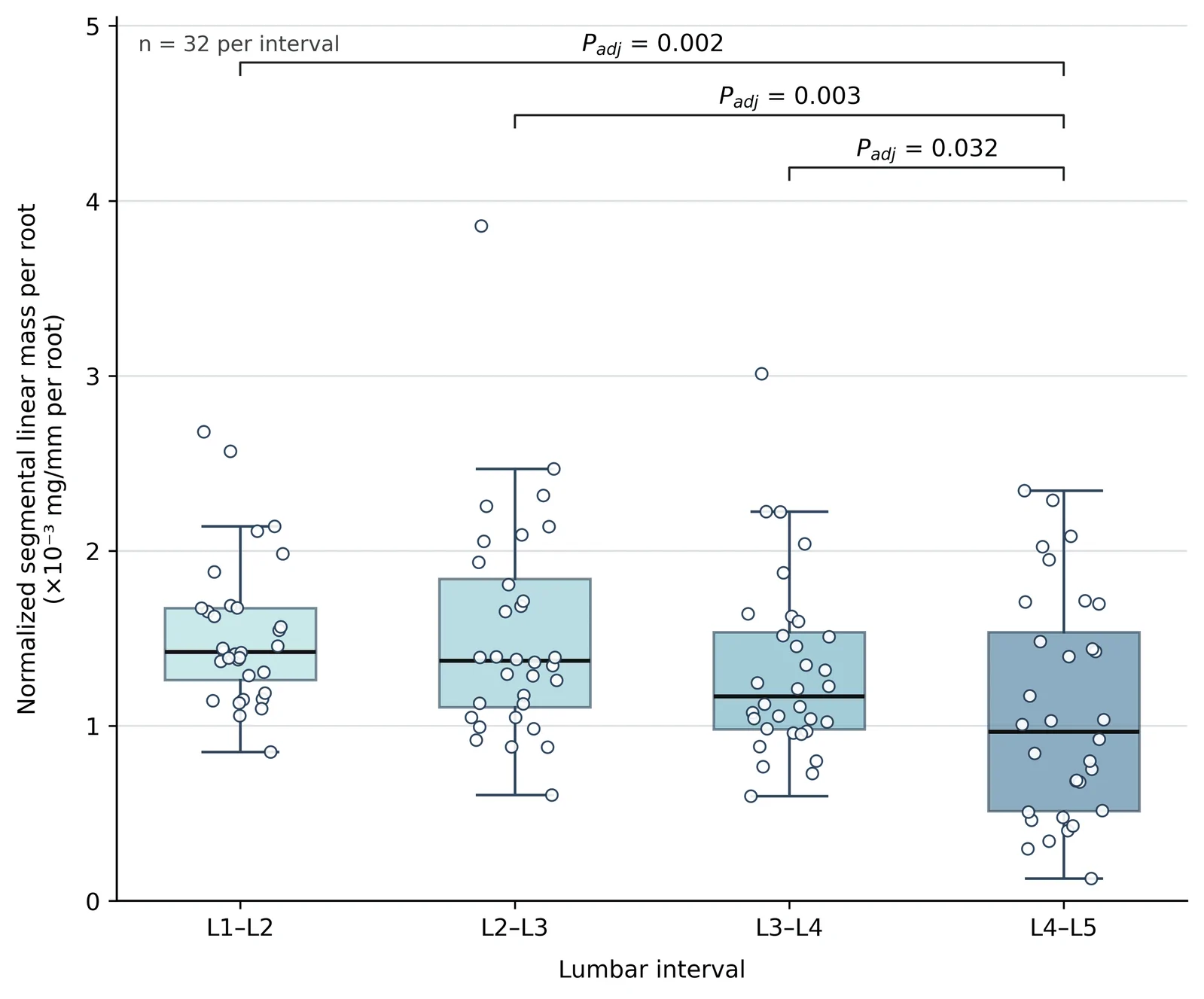
Distribution of normalized segmental linear mass per root across lumbar intervals Boxes represent the median and interquartile range; whiskers extend to 1.5 times the interquartile range, and individual points represent the 32 donor bodies. L4-L5 values were significantly lower than L1-L2, L2-L3, and L3-L4 after Bonferroni correction. Adjusted p*-*values reflect Bonferroni correction across six pairwise comparisons.

Shapiro-Wilk testing demonstrated significant deviations from normality at L1-L2, L2-L3, and L3-L4 (all p < 0.01), but not at L4-L5 (p = 0.066). Normality testing by lumbar level is summarized in Table 3.

**Table 3:** Shapiro-Wilk tests of normality for normalized segmental linear mass per root Normalized segmental linear mass per root weight deviated significantly from normality at L1-L2, L2-L3, and L3-L4 (all p < 0.01), but not at L4-L5 (p = 0.066). Analyses included 32 donor specimens.

Lumbar level	Shapiro-Wilk W	p-value
L1-L2	0.91	0.0090
L2-L3	0.87	0.0010
L3-L4	0.89	0.0030
L4-L5	0.94	0.066

A Friedman test identified a statistically significant difference in individual normalized segmental linear mass per root across lumbar levels (χ²(3) = 24.30, p = 2.16 × 10⁻⁵, Kendall’s W = 0.25). Post hoc Wilcoxon signed-rank tests with Bonferroni correction demonstrated significant differences between L1-L2 and L4-L5 (adjusted p = 0.0017), L2-L3 and L4-L5 (adjusted p = 0.0031), and L3-L4 and L4-L5 (adjusted p = 0.032). The remaining comparisons were not statistically significant. Pairwise lumbar-level comparisons are presented in Table 4.

**Table 4:** Pairwise Wilcoxon signed-rank comparisons of normalized segmental linear mass per root across lumbar levels Bonferroni adjustment was applied across six pairwise comparisons. Bonferroni-adjusted p-values < 0.05 were considered statistically significant. Paired Cohen’s d values are reported as absolute magnitudes. The effective sample size (n) represents the number of nonzero paired differences included in each comparison; zero paired differences were excluded from the Wilcoxon signed-rank calculation.

Comparison	n	Wilcoxon T	Unadjusted p	Paired Cohen’s d	Bonferroni-adjusted p
L1-L2 vs L2-L3	32	237	0.63	0.00	1.00
L1-L2 vs L3-L4	32	132	0.01	0.44	0.075
L1-L2 vs L4-L5	32	79	<0.001	0.75	0.0017
L2-L3 vs L3-L4	32	141	0.021	0.45	0.12
L2-L3 vs L4-L5	31	86	<0.001	0.68	0.0031
L3-L4 vs L4-L5	31	118	0.0054	0.53	0.032

Age was inversely correlated with mean normalized segmental linear mass per root (ρ = -0.40, p = 0.024), but this association did not remain statistically significant after FDR correction (FDR-adjusted p = 0.07). No age-related correlation at an individual lumbar level remained statistically significant after FDR correction. Age-related correlations by lumbar level are summarized in Table 5.

**Table 5:** Association between donor age and normalized segmental linear mass per root Spearman correlation analysis was used to evaluate associations between donor age and normalized segmental linear mass per root. None of the associations remained statistically significant after FDR correction (all FDR-adjusted p > 0.05). ρ, Spearman correlation coefficient; FDR, false discovery rate.

Outcome	Spearman ρ	p-value	FDR-adjusted p-value
L1-L2	-0.391	0.03	0.07
L2-L3	-0.344	0.05	0.08
L3-L4	-0.195	0.29	0.29
L4-L5	-0.333	0.06	0.08
Mean normalized segmental linear mass per root	-0.398	0.02	0.07

Donor height was positively correlated with normalized segmental linear mass per root across lumbar levels, with the strongest association observed at L3-L4 (ρ = 0.56, p = 0.01). However, no association remained statistically significant after FDR correction. Associations between donor height and normalized segmental linear mass per root are summarized in Table 6.

**Table 6:** Association between donor height and normalized segmental linear mass per root The L3-L4 association was statistically significant before adjustment (p = 0.01) but not after Benjamini-Hochberg FDR correction (FDR-adjusted p = 0.06). No association remained statistically significant after correction. Analyses included 19 of 32 donor bodies (59.4%) with complete height data. ρ, Spearman correlation coefficient; FDR, false discovery rate.

Outcome	n	Spearman ρ	Unadjusted p	FDR-adjusted p
L1-L2	19	0.21	0.40	0.40
L2-L3	19	0.35	0.15	0.18
L3-L4	19	0.56	0.01	0.06
L4-L5	19	0.41	0.08	0.14
Mean normalized segmental linear mass per root	19	0.45	0.05	0.13

Donor body weight was positively correlated with normalized segmental linear mass per root across lumbar levels; however, none of these associations were statistically significant before or after FDR correction. Associations between donor body weight and normalized segmental linear mass per root are summarized in Table 7.

**Table 7:** Association between donor body weight and normalized segmental linear mass per root No statistically significant associations were observed before or after Benjamini-Hochberg FDR correction (all FDR-adjusted p > 0.05). Analyses included 19 of 32 donor bodies (59.4%) with complete body-weight data. ρ, Spearman correlation coefficient; FDR, false discovery rate.

Outcome	n	Spearman ρ	Unadjusted p	FDR-adjusted p
L1-L2	19	0.15	0.54	0.54
L2-L3	19	0.18	0.46	0.54
L3-L4	19	0.39	0.10	0.24
L4-L5	19	0.31	0.20	0.33
Mean normalized segmental linear mass per root	19	0.41	0.08	0.24

Sex-based comparisons demonstrated higher normalized segmental linear mass per root in males than in females at all lumbar levels. Differences at L4-L5 and in normalized segmental linear mass per root were statistically significant before adjustment but did not remain significant after FDR correction. Sex-based inferential comparisons are presented in Table 8.

**Table 8:** Sex-based comparisons of normalized normalized segmental linear mass per root Differences at L4-L5 and in normalized segmental linear mass per root were statistically significant before adjustment (p = 0.02 and p = 0.03, respectively), but neither remained statistically significant after Benjamini-Hochberg FDR correction (both FDR-adjusted p = 0.08). The analysis included 18 male donor bodies (56.3%) and 14 female donor bodies (43.8%). FDR, false discovery rate.

Outcome	Mann-Whitney U	Unadjusted p	FDR-adjusted p
L1-L2	144	0.51	0.51
L2-L3	169	0.11	0.15
L3-L4	168	0.12	0.15
L4-L5	188	0.02	0.08
Mean normalized segmental linear mass per root	183	0.03	0.08

Descriptive normalized segmental linear mass per root by donor sex is presented in Table 9.

**Table 9:** Normalized segmental linear mass per root by donor sex Values are presented as the mean (standard deviation) and expressed as ×10⁻³ mg/mm per root. The analysis included 18 male donor bodies (56.3%) and 14 female donor bodies (43.8%).

Sex	L1-L2	L2-L3	L3-L4	L4-L5
Female	1.40 (0.30)	1.30 (0.40)	1.10 (0.40)	0.80 (0.50)
Male	1.60 (0.50)	1.70 (0.70)	1.50 (0.60)	1.30 (0.60)

## Discussion

Rostrocaudal variation in segmental nerve root mass

Normalized segmental linear mass per root differed significantly across the examined lumbar intervals and was lowest at L4-L5. After correction for multiple comparisons, L4-L5 values were significantly lower than those at L1-L2, L2-L3, and L3-L4. Prior cadaveric investigations have demonstrated an organized intrathecal arrangement of cauda equina roots and level-specific variation in root diameter and rootlet number [24,25]. Hogan similarly demonstrated variation in individual thoracic and lumbosacral nerve root size using a mass-to-length method [26]. The present findings extend this morphometric literature by describing normalized segmental linear mass per root across sequential lumbar vertebral intervals. Because compression, imaging, and clinical outcomes were not evaluated, the physiologic significance of this pattern remains unknown.

Radiologic and procedural implications

Our findings also highlight potential implications for radiologic interpretation and procedural practice. Whether the lower normalized segmental linear mass per root observed at caudal levels contributes to normal variability in the appearance of the cauda equina on imaging remains unknown because this study did not directly evaluate imaging. In our cohort, normalized segmental linear mass per root at L4-L5 was significantly lower than at all other lumbar levels. Although the L3-L4 and L4-L5 interspaces are commonly used for lumbar puncture and spinal anesthesia [19,20], this study did not evaluate procedural safety or direct nerve root injury. These data may inform future imaging-based studies aimed at distinguishing normal anatomic variation from true pathologic crowding.

The observed inverse associations between age and normalized segmental linear mass per root warrant further investigation. Previous studies have documented age-related neuronal decline [27]; however, the observed associations did not remain statistically significant after FDR correction, and this study cannot determine whether age-related neuronal changes explain the observed pattern. Therefore, the present findings do not support age-specific normal values. Together, these data support further study of whether age-specific considerations should be incorporated into imaging interpretation and procedural planning.

Extending current models of lumbar spinal vulnerability

Prior literature has focused on lumbar spinal vulnerability in the context of reductions in spinal canal diameter due to compressive forces, such as disc herniations or ligamentous hypertrophy [8,9]. This approach, while evidently accurate, focuses primarily on canal dimensions and external compression without directly accounting for variability in the canal’s neural contents. The findings of this study address this gap by demonstrating that normalized segmental linear mass per root varies by lumbar level. By providing direct anatomic quantification of cauda equina organization, this study provides an additional anatomical measure for future models of lumbar spinal vulnerability.

Compressive spinal pathologies and demographics

Our data showed that no statistically significant associations between normalized segmental linear mass per root and donor height, body weight, or sex remained after FDR correction. Age was inversely associated with normalized segmental linear mass per root at L1-L2 and with mean normalized segmental linear mass per root before FDR correction; however, neither association remained statistically significant after correction. Therefore, these findings provide baseline anatomical data for future studies evaluating whether intrinsic cauda equina anatomy relates to the available space within the spinal canal or its response to compressive pathology.

Broader implications

From a broader perspective, normalized segmental linear mass per root demonstrated a level-specific pattern in this donor sample, and no associations with demographic variables remained statistically significant after FDR correction. These findings provide exploratory anatomical data for future studies evaluating normal variation and its potential relationship to lumbar imaging findings [25].

Limitations and future directions

This was a single-center donor-body study with a relatively small convenience sample (n = 32). Additionally, donor height and body weight were available for only 19 (59.4%) of 32 donor bodies. No a priori power calculation was performed because no published estimates of normalized segmental linear mass per root were available and the sample size was determined by donor-body availability.

While careful planning was conducted prior to measurement, normalized segmental linear mass per root was calculated using measured segment lengths and estimated root counts rather than direct counting of the roots traversing each interval. Individual variation in conus position and nerve root anatomy may therefore have introduced measurement error. Tissue preservation, removal of connective tissue, and residual surface moisture may also have affected the recorded masses. Repeated-measurement and interobserver reliability were not evaluated.

The observed age-related trends warrant further investigation and validation in larger, multicenter studies. Future studies incorporating direct root counting, standardized tissue preparation, repeated measurements, and three-dimensional or volumetric methods may help refine normative ranges and improve the overall validity of these findings. Because imaging and clinical outcomes were not evaluated, future studies should directly examine whether these anatomical measurements correspond to imaging appearance or clinical pathology.

## Conclusions

Lumbar spinal vulnerability may reflect a complex interplay between external compressive forces and the intrinsic organization of the cauda equina within the spinal canal. In this study, normalized segmental linear mass per root generally decreased from rostral to caudal levels and was lowest at L4-L5. No associations with sex, age, height, or body weight remained statistically significant after correction for multiple comparisons. Together, these findings provide exploratory baseline data regarding the anatomic organization of the cauda equina and a foundation for future imaging and clinical correlation studies.
